# Nonreciprocal second harmonic generation in a magnetoelectric material

**DOI:** 10.1126/sciadv.abe2793

**Published:** 2021-04-16

**Authors:** Shingo Toyoda, Manfred Fiebig, Taka-hisa Arima, Yoshinori Tokura, Naoki Ogawa

**Affiliations:** 1 RIKEN Center for Emergent Matter Science (CEMS), Saitama 351-0198, Japan.; 2 Department of Materials, ETH Zurich, 8093 Zurich, Switzerland.; 3 Department of Advanced Materials Science, University of Tokyo, Kashiwa 277-8561, Japan.; 4 Tokyo College, University of Tokyo, Tokyo 113-8656, Japan.; 5 Department of Applied Physics, University of Tokyo, Tokyo 113-8656, Japan.; 6 PRESTO, Japan Science and Technology Agency (JST), Kawaguchi 332-0012, Japan.

## Abstract

Mirror symmetries are of particular importance because they are connected to fundamental properties and conservation laws. Spatial inversion and time reversal are typically associated to charge and spin phenomena, respectively. When both are broken, magnetoelectric cross-coupling can arise. In the optical regime, a difference between forward and backward propagation of light may result. Usually, this nonreciprocal response is small. We show that a giant nonreciprocal optical response can occur when transferring from linear to nonlinear optics, specifically second harmonic generation (SHG). CuB_2_O_4_ exhibits SHG transmission changes by almost 100% upon reversal of a magnetic field of just ±10 mT. The observed nonreciprocity results from an interference between magnetic-dipole and electric-dipole SHG. Although the former is inherently weaker than the latter, a resonantly enhanced magnetic-dipole transition has a comparable amplitude as a nonresonant electric-dipole transition, thus maximizing the nonreciprocity. Multiferroics and magnetoelectrics are an obvious materials platform to exhibit nonreciprocal nonlinear optical functionalities.

## Teaser

Nearly perfectly unidirectional second harmonic generation of light is realized in a magnetoelectric material.

## INTRODUCTION

Unidirectional manipulation of photons is a key issue in modern information technology as exemplified by optical isolators used for lasers and in optical networks. While conventional optical isolators are the composite of a magneto-optical medium, magnets, and polarizers, recent studies show that such a one-way flow of photons can be achieved in single bulk magnetoelectric materials, where time-reversal and space-inversion symmetries are simultaneously broken. Such nonreciprocal propagation occurs because the optical properties change with the reversal of the propagation direction of light $\vec{k}$ (*1*–*3*). In the past, a wide variety of nonreciprocal phenomena has been found in linear optics, including the absorption (*4*–*7*), emission (*8*, *9*), refraction (*10*), and diffraction (*11*) of light. In stark contrast, there have been few reports on nonreciprocal phenomena in the nonlinear optical regime (*12*, *13*), although they are naturally expected (*1*).

**Fig. 1:**
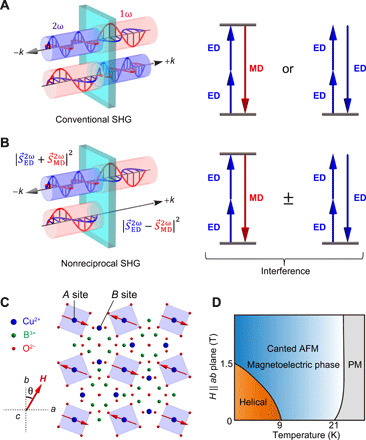
Schematic illustration of conventional SHG and nonreciprocal SHG. (**A**) SHG intensity generally remains unchanged after reversal of the propagation direction of the light because the SHG signal originates from either ED-SHG or MD-SHG. The electronic transition processes for resonant MD-SHG and nonresonant ED-SHG are sketched on the right-hand side. (**B**) When the ED-SHG interferes with MD-SHG, the total intensity can depend on the propagating direction of the light. In particular, when the ED-SHG and the MD-SHG yield become similar in both amplitude and phase, their constructive interference substantially enhances the total SHG signal for light propagating in one particular direction. The situation reverses for the opposite propagation direction due to destructive interference, resulting in the suppression or even extinction of the SHG output. (**C**) Crystal and magnetic structures of CuB_2_O_4_ in the canted antiferromagnetic phase, projected onto the (001) plane. The red arrows indicate the magnetic moments of the Cu^2+^ holes in a magnetic field μ_0_*H* ⊥ [001]. (**D**) Magnetic phase diagram of CuB_2_O_4_ within a magnetic field along the *ab* plane. Canted AFM and PM represent the canted antiferromagnetic and paramagnetic phases, respectively.

Second harmonic generation (SHG), which denotes the frequency-doubling of a light wave in a material, is one of the simplest nonlinear optical processes. SHG can be classified into two types depending on its origin. One is electric-dipole (ED)–SHG, in which the frequency-doubled electric polarization ${\vec{P}}_{i}(2\omega )$ in a material is considered, while the other is magnetic-dipole (MD)-SHG, which refers to a frequency-doubled magnetization ${\vec{M}}_{i}(2\omega )$ (Fig. 1A). Here, we neglect electric quadrupole contributions because they are forbidden for the *d-d* transition of Cu^2+^ holes in CuB_2_O_4_ (*4*). The source term $\vec{S}(2\omega )$of these SHG processes is given by (*14*)

$$\begin{array}{cc} \vec{S}(2\omega ) & =\mu_{0}\frac{\partial^{2}\vec{P}(2\omega )}{\partial t^{2}}+\mu_{0}(\nabla \times \frac{\partial \vec{M}(2\omega )}{\partial t})\\ & =-4\omega^{2}\mu_{0}\vec{P}(2\omega )+4\omega \mu_{0}\vec{k}\times \vec{M}(2\omega ) \end{array}$$ (1)

The SHG intensity on the detector will be $I(2\omega )\propto {|\vec{S}(2\omega )|}^{2}$, indicating that the intensity changes with the reversal of $\vec{k}$ when both processes coexist and interfere with each other (Fig. 1B). Obviously, for a given $\vec{k}$, if the first term (ED-SHG) has the same amplitude and phase as the second term (MD-SHG), the total intensity *I*(2ω) is enhanced by constructive interference, but can be extinguished just by the reversal of $\vec{k}$. Note that we can also control the SHG interference by reversing $\vec{P}(2\omega )$or $\vec{M}(2\omega )$, which, for example, can be realized by the reversal of the static magnetization $\vec{M}$ of the system with an external magnetic field. In the present study, ED-SHG is an *i*-type tensor and MD-SHG is a *c*-type tensor (see the Supplementary Materials); the latter changes sign under the reversal of the magnetic field. However, experimental realization of such a huge modulation of the SHG yield (i.e., nonreciprocal SHG of ~100% efficiency) remains a challenge. ED-SHG usually dominates MD-SHG because in the expansion of the electromagnetic field potential, with respect to $\vec{k}$, the two terms are of zeroth and first order, respectively. Therefore, MD-SHG is less commonly observed because usually it is visible in centrosymmetric materials only, where ED-SHG is forbidden or permitted only through inversion symmetry–breaking magnetically ordered phases (*12*, *13*, *15*). This unbalance in the ED and MD transition dipole moments hampers the manifestation of large optical nonreciprocity.

Here, we report on the observation of nonreciprocal SHG in noncentrosymmetric CuB_2_O_4_, where the SHG intensity changes by 97% upon the reversal of an external magnetic field. The effect is most pronounced for light around 1.4 eV, which is resonant with the lowest intra-atomic *d-d* transition of the Cu^2+^ ions. We found that the MD-SHG process is resonantly enhanced at this *d-d* transition, in contrast to the ED-SHG process. The resultant resonant MD-SHG and nonresonant ED-SHG contributions are of the same order of magnitude, yielding a large nonreciprocal signal via their interference.

CuB_2_O_4_ crystalizes in a noncentrosymmetric tetragonal structure with the space group symmetry *I*$\bar{4}$2*d* (point group $\bar{4}$2m) (*16*). The Cu^2+^ (*d^9^*, *S* = 1/2) ions occupy two inequivalent sites denoted as *A* and *B* (Fig. 1C), where Cu^2+^ ions on the *A* site are responsible for the linear nonreciprocal optical properties (*4*). This material undergoes successive magnetic transitions at *T*_N_ = 21 K and T* = 9 K (*17*, *18*) (see Fig. 1D). Below T*, magnetic moments at both *A* and *B* sites exhibit incommensurate helical order. Between *T*_N_ and T*, magnetic moments of the Cu^2+^ ions show an easy-plane-type canted antiferromagnetic order, as schematically shown in Fig. 1D. By the application of an in-plane external magnetic field of the order of 10 mT, we can align the in-plane magnetization and obtain a single-domain state. In the canted antiferromagnetic phase, where the time-reversal and space-inversion symmetries are simultaneously broken, CuB_2_O_4_ shows a magnetoelectric effect, which is explained by the modification of the metal-ligand hybridization with the Cu^2+^ moments (*19*, *20*). The magnetic-field-induced electric polarization appears along the *c* axis (*P* ∝ sin 2θ) in an external magnetic field in the *ab* plane, where θ denotes the angle between the crystal’s [010] axis and the direction of the magnetic field (*20*).

## RESULTS AND DISCUSSION

**Fig. 2:**
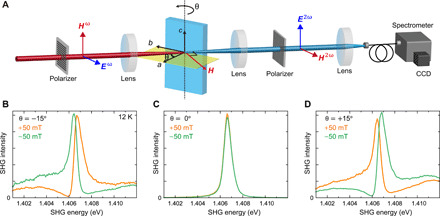
Experimental observation of nonreciprocal SHG. (**A**) Optical setup to detect nonreciprocal SHG emission. The light pulses (photon energy ℏω = 0.703 eV) from an optical parametric amplifier were focused onto a CuB_2_O_4_ single crystal after setting their polarization to *E*^ω^ ⊥ [001] and *H*^ω^ ∥ [001] by using a Glan-laser prism. The sample was tilted around the *c* axis by an angle θ. SHG spectra for linearly polarized light (*E*^2ω^ ∥ [001] and *H*^2ω^ ⊥ [001]) were measured in a transmission geometry with a spectrometer and a charge-coupled device camera as detector. An external magnetic field was applied in the *ab* plane normal to the propagation direction of the light (Voigt geometry). (**B** to **D**) Spectral dependence of the SHG for tilt angles (B) θ = −15°, (C) θ = 0°, and (D) θ = +15°, measured at *T* = 12 K. Green and yellow lines show the spectra in a magnetic field of μ_0_*H* = −50 mT and μ_0_*H* = +50 mT, respectively.

Figure 2A shows the experimental setup to detect the nonreciprocal SHG signal. The SHG intensity was measured in a transmission geometry in Voigt configuration (external magnetic field $\vec{H}⊥\vec{k}$). The light source was a regenerative amplifier, which produced 190-fs laser pulses at 6 kHz. The energy of the fundamental light was tuned by an optical parametric amplifier (OPA) to ℏω = 0.703 eV, for which the SHG energy 2ℏω = 1.406 eV was resonant with the *d-d* transition of Cu^2+^ holes between the *d_x2-y2_* and *d_xy_* orbitals (*4*, *18*, *21*–*23*). Note that the spectral width of the electronic transition of ~1 meV was one order of magnitude smaller than the energy spread of the incident laser pulse. We used a spectrometer to resolve the fine structure of the SHG spectrum. The thickness of the sample was 50 μm, with the widest crystal faces exhibiting (100) orientation. The sample was mounted on a copper holder, which could rotate around the *c* axis by the angle θ (Fig. 2A). We tilt the sample around the *c* axis, and therefore, the electric field of the fundamental light *E*^ω^ is always in the *ab* plane and that of the SHG field *E*^2ω^ remains parallel to the *c* axis. This suggests that the refractive index and, hence, the phase matching condition are not affected by the tilting of the sample because the crystal structure of CuB_2_O_4_ is uniaxial. Figure 2 (B to D) shows the tilt-angle dependence of the SHG contribution polarized along the *c* axis in a magnetic field μ_0_*H* of 50 mT. When the sample is not tilted (θ = 0°), the electric polarization is absent for *H* parallel to the [010] axis (*20*). In this situation, the SHG signal originates solely from MD-SHG, because the complementing ED-SHG contribution with *E*^ω^ ∥ [010] is zero (see the Supplementary Materials). This results in the absence of ED-MD interference and, hence, of a change in SHG intensity upon the reversal of the magnetic field (see Fig. 2C). When the sample is tilted around the *c* axis, the [100] component of the electric field of light ${\vec{E}}_{a}(\omega )$ and the associated ED-SHG from χ*_cab_* and χ*_cba_* become allowed. Figure 2 (B and D) shows the SHG spectra in the magnetic field μ_0_*H* = 50 mT for the sample tilted by θ = −15° and θ = +15°, respectively. The associated SHG spectra exhibit drastic changes with the reversal of magnetic field, demonstrating the nonlinear optical nonreciprocity. Notably, the SHG spectra show a Fano resonance–like asymmetric shape. A Fano resonance is a signature of the interference between a resonant process and a nonresonant background (*24*, *25*), in the present case represented by MD-SHG for the former and ED-SHG for the latter.

**Fig. 3:**
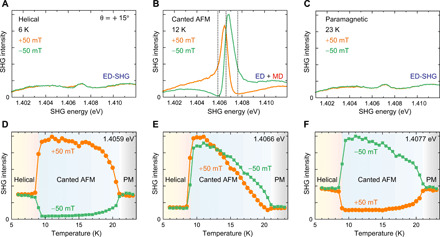
Temperature dependence of nonreciprocal SHG. SHG spectra measured at (**A**) 6 K in the helical, (**B**) 12 K in the canted AFM, and (**C**) 23 K in the paramagnetic phase with the sample tilted by θ = +15°. Green and yellow lines indicate the spectra in a magnetic field of μ_0_*H* = −50 mT and μ_0_*H* = +50 mT, respectively. The magnetic field is applied in the *ab* plane in the Voigt geometry (see Fig. 2A). (**D** to **F**) Temperature dependence of the SHG intensities (D) below the resonance energy (Δ = −0.7 meV), (E) at the resonance energy (Δ = 0 meV), and (F) above the resonance energy (Δ = +1.1 meV), where Δ denotes the energy deviation from the resonance energy 1.4066 eV. These photon energies are indicated by the dashed lines in (B).

Next, we investigate the temperature dependence of the nonreciprocal SHG signal for the sample tilted by θ = + 15°. Figure 3 (A to C) shows the SHG spectra at different temperatures in a magnetic field μ_0_*H* = ± 50 mT. Whereas the nonreciprocal signal is clearly observed in the canted antiferromagnetic phase, it disappears in the helical and the paramagnetic phases, where the time-reversal symmetry is preserved. This result confirms that the space- and time-inversion symmetry breaking is essential to the emergence of the nonreciprocal behavior. The SHG signal shows a broad and featureless spectral distribution in the helical and paramagnetic phases, which, referring to our analysis of the Fano-resonant behavior above, points to its ED-SHG origin. ED-SHG can be expressed by ${\vec{P}}_{i}(2\omega )=\epsilon_{0}\chi_{\mathrm{ijk}}^{\text{ED}}{\vec{E}}_{j}(\omega ){\vec{E}}_{k}(\omega )$, where $\chi_{\mathrm{ijk}}^{\text{ED}}$ and $\vec{E}(\omega )$ are the SHG susceptibility and the electric field of the incident light, respectively. The $\bar{4}$2m point-group symmetry allows the SHG tensor components χ*_cab_* and χ*_cba_* for SHG light polarized along the *c* axis (*18*, *26*), which becomes accessible in our experiment, where *E*^ω^ ⊥ [001] and *E*^2ω^ ∥ [001]. The ED-SHG polarization ${\vec{P}}_{c}(2\omega )$ associated to χ*_cab_* and χ*_cba_* changes sign with the change of rotation of the sample from +15° to −15° because of the associated reversal in the sign of ${\vec{E}}_{a}(\omega )$. This explains the sign reversal in the magnetic-field dependence in Fig. 2 (B and D).

Figure 3 (D to F) shows the temperature dependence of the SHG intensity at photon energies of 1.4059, 1.4066, and 1.4077 eV, which are indicated by the dashed lines in Fig. 3B. At off-resonant photon energies (Fig. 3, D and F), the ED-MD interference leads to a pronounced nonreciprocal signal in the canted antiferromagnetic phase. In contrast, the SHG intensity shows little change with the reversal of magnetic field at the resonant energy (see Fig. 3E). Here, we defined the resonance energy as the peak position of the pure MD-SHG spectrum in Fig. 2C, which is measured at *T* = 12 K without tilting the sample (θ = 0°). The remaining dependence on the direction of the magnetic field is caused by the small shift of the resonance energy experiences with temperature (*21*). Thus, at the resonance energy, the nonreciprocity of the signal in the canted antiferromagnetic phase formally disappears.

**Fig. 4:**
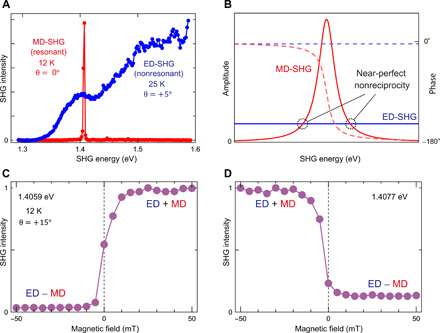
Origin of nonreciprocal SHG. (**A**) Spectra of the MD-SHG and ED-SHG contributions. The MD-SHG spectrum is obtained without tilting the sample (θ = 0°) in a magnetic field of μ_0_*H* = +50 mT along the [010] axis at *T* = 12 K in the canted AFM phase. The ED-SHG spectrum is measured at *T* = 25 K in the paramagnetic phase for a tilt angle θ = 5°. Both spectra are normalized to the maximum values. (**B**) Schematic of the origin of maximum nonreciprocity. Blue and red lines (dashed lines) show the amplitude (phase) of ED-SHG and MD-SHG contributions, respectively. The resonantly enhanced MD-SHG contribution shows a comparable amplitude as the nonresonant ED-SHG contribution and has the same amplitude and the same (the opposite) phase below (above) the resonant photon energy as indicated by dotted circles. (**C** and **D**) Magnetic-field dependence of the SHG intensity (*T* = 12 K) for the sample tilted by +15° (C) below (1.4059 eV) and (D) above (1.4077 eV) the resonance energies. These photon energies are indicated by dashed circles in (B).

To further elucidate the origin of the nonreciprocal signal, we investigate the SHG spectrum across a broader spectral range (see Fig. 4A). The ED- and MD-SHG spectra can be measured separately by properly selecting the temperature and the tilt angle θ. Pure ED-SHG is obtained at 25 K in the paramagnetic phase for the sample tilted by 5°, whereas pure MD-SHG is measured at 12 K in the canted antiferromagnetic phase without tilting the sample. The ED-SHG signal does not show any peak at the *d-d* transition of 1.4 eV and increases with small variations as the photon energy is raised. We ascribe this to the nonresonant virtual excitation of the charge transfer transition between Cu^2+^ and O^2−^ ions. On the other hand, the MD-SHG signal is resonantly enhanced at the *d-d* transitions between the *d_x2-y2_* and *d_xy_* orbitals of Cu^2+^ holes at the *A* site. These results explain why MD- and ED-SHG can be of comparable amplitude, which then leads to the strongly nonreciprocal signal.

Next, we discuss the observed spectral shape. Figure 4B illustrates a schematic of the MD- and ED-SHG susceptibilities around the resonance energy. Both the amplitude and phase of the ED-SHG contribution are almost unchanged around the resonance, because its origin is unrelated to the *d-d* transitions. On the other hand, phase and amplitude of the MD-SHG contribution show pronounced changes in this region. The MD-SHG wave experiences a 180° phase shift across the resonance energy and the amplitude sharply increases to show the resonance peak. Therefore, the ED- and MD-SHG light fields interfere constructively (destructively) at photon energies below (above) the MD resonance, which explains the sign change of the nonreciprocal effect across the resonance. Notably, the amplitudes of the MD- and ED-SHG contributions become the same at the positions indicated by dotted circles in Fig. 4B. Here, we expect a maximum of the nonreciprocal behavior in the SHG response by the ED-MD interference. Figure 4C shows the magnetic-field dependence of the SHG intensity at 1.4059 eV (that is, slightly below the resonance). The SHG intensity almost disappears for the negative magnetic field, whereas a strong SHG signal shows up for the same yet positive magnetic-field value, indicating that MD-SHG and ED-SHG light waves of the same amplitude interfere. Slightly above the resonance (1.4077 eV), the situation is reversed, as shown in Fig. 4D. This is in excellent agreement with our model of the interference between resonant MD and nonresonant ED components. The asymmetry in the extinction of the SHG signal above (Fig. 4D) and below (Fig. 4C) the resonance photon energy can be explained by the phase between the ED-SHG and MD-SHG contributions. Below the resonance, ED- and MD-SHG are in phase, which allows for near-perfect nonreciprocity. However, since the phase change of the MD-SHG contribution across the resonance is less than 180°, the phase between ED- and MD-SHG slightly differs above the resonance, resulting in the imperfect interference and, hence, extinction of the SHG in Fig. 4D.

To conclude, we experimentally demonstrate the nearly perfectly unidirectional propagation of SHG light waves in CuB_2_O_4_. The SHG intensity changes by almost 100% upon the reversal of a small magnetic field of 10 mT. The observed nonreciprocity originates from the interference between ED-SHG and MD-SHG. The MD-SHG process is resonantly enhanced at the *d-d* transition of Cu^2+^ holes and becomes comparable in amplitude to the nonresonant broadband ED-SHG process. This work establishes that nonreciprocal wave-propagation effects are not limited to linear optics but are also present as nonlinear optical effects and exhibit a magnitude up to the optical-diode-like unidirectional propagation of light.

## MATERIALS AND METHODS

### Sample preparation

Single crystals of CuB_2_O_4_ were grown by a flux method. Powders of CuO (7.250 g), B_2_O_3_ (15.228 g), and LiCO_3_ (4.041 g) were mixed. They were heated in air at 1020°C, subsequently cooled down to 800°C at a rate of 1.1°C/hour, and then cooled to room temperature at a rate of 16°C/hour. A single crystal was oriented by using a Laue x-ray diffractometer and cut into thin plates of 50 μm thickness with the widest (100) faces. The sample surfaces were specularly polished by alumina lapping films.

*Note added in proof*: We have recently become aware of the related work by J. Mund *et al*. (arXiv: 2005.05393 2020).

## SUPPLEMENTARY MATERIALS

Supplementary material for this article is available at http://advances.sciencemag.org/cgi/content/full/7/16/eabe2793/DC1
